# Hyperspectral image classification network based on multiscale spatial-spectral fusion and semantic enhancement encoder

**DOI:** 10.1038/s41598-026-46407-y

**Published:** 2026-04-25

**Authors:** Jia Yu, Bailian Tang, Cheng Zha, Nan Jiang, Ruikang Liu, Tiwei Zeng, Zhen Ye, Fan Feng, Chongyi Zhuang

**Affiliations:** 1 School of Information and Software Engineering, East China Jiaotong University, Nanchang, 330013 China; 2 Jiangxi Shengtai Precision Optics Co., Ltd., Xinyu, 336600 China; 3 Beihai Internet Information Security Center, Beihai, 536006 China; 4 Lishui University, Lishui, 323000 China

**Keywords:** Environmental sciences, Mathematics and computing, Optics and photonics

## Abstract

Hyperspectral image classification has become a pivotal task in remote sensing data processing. However, current deep learning-based methods still face challenges in effectively extracting discriminative joint spatial-spectral features, especially when handling complex spatial structures and highly variable spectral responses. To address this issue, a novel hyperspectral image classification network (MSNet) based on multiscale spatial-spectral fusion (MSSF) and semantic enhancement encoder (SEE) is proposed. First, principal component analysis is employed to reduce the dimensionality of hyperspectral image, preserving essential spectral information while mitigating noise interference. Second, the proposed MSSF extracts multiscale spatial features through a dedicated spatial branch while simultaneously mining discriminative spectral information via a parallel spectral branch. Feature fusion is subsequently employed to achieve synergistic interaction between spatial and spectral representations, effectively capturing joint characteristics of land covers across multiple scales. Third, the designed SEE incorporates a multi-head attention mechanism to explicitly model global feature dependencies, thereby enhancing the representation of semantically critical regions. Extensive experiments have been conducted on the Pavia University and Salinas datasets. Experimental results demonstrate that MSNet achieves outstanding performance, with overall accuracy reaching 95.68% and 96.84%, respectively, surpassing existing mainstream methods and validating its effectiveness.

**Supplementary Information:**

The online version contains supplementary material available at 10.1038/s41598-026-46407-y.

## Introduction

Hyperspectral Image (HSI) constitutes a three-dimensional data cube acquired by imaging spectrometers across the visible to short-wave infrared spectral range, wherein each pixel contains spectral information from hundreds of contiguous narrow bands with exceptionally high spectral resolution. This unique spatio-spectrally integrated data structure simultaneously captures two-dimensional spatial distribution characteristics of surface features and their continuous spectral response properties, thereby forming spectrally distinctive feature vectors capable of material identification. As a research hotspot in the remote sensing field, HSI processing has developed multiple technical branches, among which hyperspectral anomaly detection^1,2^ and HSI classification^3^ are the most representative. Hyperspectral anomaly detection, as an unsupervised intelligent interpretation technique, can automatically identify targets that exhibit spectral differences from the surrounding background without any prior spectral information. HSI classification refers to the process of categorizing land cover types at either pixel-level or object-level scales by leveraging high-dimensional spectral feature spaces and employing pattern recognition or machine learning algorithms on remote sensing imagery. This technique demonstrates unique application value in domains such as fine-grained land classification^4^, and crop species identification^5^, providing critical data support and technical means for scientific decision-making in smart agriculture, geological surveys, environmental monitoring, and related fields.

Traditional HSI classification methods^6,7^ primarily rely on pixel-level spectral features, utilizing statistical learning^8^ or machine learning^9^ algorithms to model the spectral response patterns of ground objects. These approaches achieve category discrimination by constructing discriminant functions. Although such methods demonstrate stable performance and high computational efficiency in specific scenarios, they suffer from inherent limitations in feature representation, including insufficient utilization of spatial information and limited capability in extracting nonlinear features. These drawbacks often lead to constrained classification accuracy in complex land cover scenarios. With the continuous advancement of deep learning, Convolutional Neural Networks (CNNs)^10^ have enabled joint extraction of spatial and spectral features through local receptive fields^11^, giving rise to various typical network architectures in HSI classification, such as 2D-CNN and 3D-CNN^12^.

CNN-based methods primarily rely on convolutional operations within sliding windows for feature extraction. However, the limited receptive field of fixed-size convolutional kernels restricts feature capture to local spatial-spectral neighborhoods. This localized feature extraction mechanism leads to a fundamental limitation in modeling long-range dependencies within the data. Transformer architecture^13^ enables dynamic global context modeling through self-attention mechanisms, adaptively establishing feature correlations among pixels at arbitrary distances. This capability provides a more powerful feature learning framework for HSI classification. He et al.^14^ proposed the spatial-spectral transformer, which tackles classification by extracting local spatial features via a meticulously designed CNN, modeling spectral dependencies with an modified Transformer, and employing a multilayer perceptron^15^ for final decision-making. Zhang et al.^16^ proposed a lightweight transformer network, which incorporates a channel lightweight multi-head self-attention module and a position lightweight multi-head self-attention module to efficiently capture global information. This method effectively addresses the issues of high computational complexity and proneness to overfitting in traditional Transformers, achieving a balance between accuracy and efficiency across multiple hyperspectral datasets.

Despite the remarkable progress achieved in existing HSI classification methods, they still face considerable challenges when dealing with complex scenarios such as spectral variability^17^ and spectral similarity^18^. Many approaches struggle to effectively capture spatial-spectral characteristics across multiple scales, lacking the ability to integrate hierarchical representations that span from local details to global contextual information. Moreover, the high-dimensional nature of HSI data, accompanied by substantial redundant and noisy spectral bands, further hinders the learning of semantically discriminative features. This limitation adversely affects the model’s capacity to generalize in distinguishing subtle inter-class variations, thereby constraining the overall classification accuracy and robustness. To address these challenges, a novel HSI classification network based on MSSF and SEE is proposed. The main contributions of this work are summarized as follows:

1. The MSSF module is proposed to comprehensively harness both spatial and spectral information within hyperspectral imagery. It incorporates dedicated spatial and spectral branches to extract and integrate discriminative features. The spatial branch extracts multiscale spatial features through a set of receptive fields, enabling the capture of contextual patterns ranging from local textures to global structures. Simultaneously, the spectral branch discriminatively mines salient spectral signatures by emphasizing informative bands and suppressing redundant ones, thereby enhancing inter-class separability.
2. The designed SEE module employs a multi-head attention mechanism to explicitly model global semantic dependencies, focusing on enhancing the contextual relevance of feature representations. By adaptively attending to semantically salient regions and reinforcing their contributions, the module effectively amplifies critical information while preserving granular details, thereby providing a more discriminative and semantically meaningful feature encoding for downstream tasks.
3. Extensive experiments have been conducted on the widely-used Pavia University dataset to thoroughly evaluate the performance of the proposed MSNet. The experimental results demonstrate that MSNet achieves outstanding classification performance, with the OA reaching 95.68%, and the K amounting to 94.33%. These metrics significantly surpass those of existing mainstream methods, thereby validating the effectiveness and superiority of the proposed method in HSI classification tasks.

The remainder of this paper is organized as follows. “Related work” section provides a review of existing HSI classification methods based on CNN and Transformer. “MSNet” section elaborates on the architecture of the proposed MSNet, detailing its core components, namely MSSF and SEE. “Experiment” section presents a comprehensive experimental analysis, including dataset descriptions, implementation details, ablation studies, and comparative results. “Conclusion and future work” section is conclusions.

## Related work

### CNN-based methods

As an important foundational framework for HSI classification, CNN has driven significant early advancements in this field due to their powerful feature representation capabilities. It can effectively learn the complex spatial and spectral features in high-dimensional HSI data, and automatically establish the mapping relationship between these features and the land cover categories, significantly improving the classification accuracy.

The demonstrated efficacy of CNN in HSI classification has spurred significant methodological developments, with numerous CNN-based methods emerging to address various aspects of HSI classification. Li et al.^19^ proposed a 3D-CNN-based HSI classification framework that directly processes raw HSI cube data without preprocessing, enabling effective extraction of deep spectral-spatial features. This method demonstrates superior performance with low model complexity, excellent training convergence characteristics, and strong generalization capability. Yang et al.^20^ designed four novel CNN models for HSI classification by integrating spatial context and spectral correlation, achieving superior performance across multiple benchmark datasets. Roy et al.^21^ proposed a Hybrid Spectral CNN (HybridSN) that effectively extracts joint spatial-spectral features through 3D-CNN while simultaneously capturing high-level spatial representations via 2D-CNN. This hybrid architecture achieves significant improvements in classification accuracy while maintaining computational efficiency through reduced model complexity. Zhu et al.^22^ proposed an en-to-end residual spectral-spatial attention network that directly process raw 3D data cubes. The network architecture employs a spectral attention module to selectively enhance discriminative spectral bands, while utilizing a spatial attention mechanism to concentrate on salient local neighborhood pixels. These attention modules are strategically embedded within a residual CNN framework, enabling adaptive feature refinement and computationally efficient training. Alkhatib et al.^23^ proposed Tri-CNN, an approach employing multi-scale 3D-CNN and three-branch feature fusion. The aforementioned research progress has conclusively demonstrated the effectiveness of CNN as a core framework for HSI classification.

### Transformer-based methods

Although CNN-based methods have demonstrated competitive performance in HSI classification, their inherent local receptive fields present fundamental limitations in effectively modeling globally distributed spectral correlations and complex spatial contexts. These limitations have motivated increasing adoption of transformer architectures, which employ self-attention mechanisms to explicitly capture long-range dependencies between pixels and adaptively model spectral-spatial interactions across different regions. Following the introduction of the Vision Transformer (ViT) by Dosovitskiy et al.^24^ for image classification, transformer-based models have been increasingly adopted in HSI classification, offering superior modeling of non-local spectral-spatial interactions compared to traditional CNNs.

Recent research in HSI has increasingly focused on transformer architectures due to their unique ability to model global relationships. While ViT have shown promise for HSI classification through global context modeling, existing patch-based approaches fail to capture true global features. To address this issue, Yan et al.^25^ proposed a hybrid convolution-ViT network that processes entire HSI images via semantic segmentation. Their approach combines an enhanced hybrid convolution and ViT module for joint local-global feature extraction, utilizing a dual-branch architecture to achieve spatial-spectral fusion and superior performance. Hong et al.^26^ proposed a novel network called SpectralFormer, which captures local spectral sequential information from adjacent bands of HSI and mitigates information loss through cross-layer skip connections. Mei et al.^27^ proposed a group-aware hierarchical transformer that introduces grouped pixel embedding to enhance local spatial-spectral relationships while maintaining global modeling capabilities through hierarchical architecture. Yang et al.^28^ proposed a Hyperspectral Image Transformer (HiT) classification network, which integrates convolutional operations into the transformer architecture to capture subtle spectral distinctions and convey local spatial contextual information. Wang et al.^29^ transformed hyperspectral data into three-channel images compatible with existing ImageNet-pretrained networks, employed transformer to encode globally aggregated spatial contexts, and ultimately integrated the segmentation results of the three spectral images into a final prediction through a voting mechanism. Transformers excel at extracting global features and can outperform other deep learning models when sufficient training data is available. However, most transformer-based HSI classifiers rely on self-attention mechanisms^30^ and multilayer perceptrons, which inherently limit their few-shot learning capability. Therefore, Li et al.^31^ proposed a lightweight self-pooling transformer, which employs a self-supervised autoencoder for dimensionality reduction, incorporates two parameter-free modules for spectral enhancement and spatial-spectral interaction, and utilizes lightweight channel embedding followed by a fully connected layer^32^ for feature extraction and classification. Sun et al.^33^ proposed a spectral-spatial feature tokenization transformer (SSFTT). This model first extracts shallow spectral-spatial features through a convolutional layer, then uses a Gaussian weighted feature tokenizer to transform these features, followed by feature learning through a Transformer encoder, and finally classifies the learnable labels through a linear layer. Zhang et al.^34^ proposed a novel transformer model named the convolutional autoencoder meets lightweight vision transformer (CAEVT). This model synergistically integrates the local feature extraction capability of a convolutional autoencoder with the global contextual modeling strength of a lightweight vision transformer. Roy et al.^35^ proposed a novel morphological transformer (morphFormer) architecture that incorporates learnable spectral and spatial morphological networks. This framework utilizes spectral and spatial morphological convolution operations integrated with an attention mechanism to enhance the interaction between structural and shape information derived from hyperspectral tokens and the classification token. To address the challenges of distinguishing land cover boundaries and the susceptibility to redundant information interference in hyperspectral image classification, Zhang et al.^36^ proposes a classification network MATNet that integrates multi-attention and Transformer. The network focuses on salient features through spatial and channel attention, introduces a Transformer encoder to extract deep semantic information, and designs an improved label smoothing loss function Lpoly to enhance model generalization. Zhao et al.^37^ designed the Groupwise Separable Convolutional Vision Transformer Network (GSCViT), which utilizes groupwise separable convolution to extract local spectral-spatial features and introduces a groupwise separable self-attention mechanism to achieve collaborative modeling of local and global spatial features. Ahmad et al.^38^ proposed WaveFormer to mitigate information loss from average pooling in Transformer-based hyperspectral image classification, employing wavelet transform for reversible downsampling to maintain data integrity, which enables efficient attention learning and yields significant accuracy improvements. Xu et al.^39^ introduced the Dual-Selective Fusion Transformer Network (DSFormer) for HSI classification. It achieves integrated spatial-spectral representation by adaptively fusing features from diverse receptive fields. This selective process mitigates redundant information and prioritizes the most salient tokens, thereby enhancing feature discriminability. While recent transformer-based methods have achieved remarkable progress in HSI classification, most of them still overly rely on spectral-spatial features at the pixel or local patch level, failing to effectively capture and leverage hierarchical semantic information across multiple scales. Furthermore, due to the inherent complexity of modeling high-level semantic concepts from limited samples, their ability to generalize under extreme few-shot conditions remains constrained. Therefore, enhancing multi-scale modeling and semantic feature representation is crucial for improving the classification performance of the model.

## MSNet

The implementation details of the proposed MSNet are elaborated in this section. The overall architecture of MSNet is first outlined, followed by a detailed explanation of its two key components, namely Multiscale Spatial-Spectral Fusion (MSSF) and Semantic Enhancement Encoder (SEE).

### Overview of MSNet

**Fig. 1:**
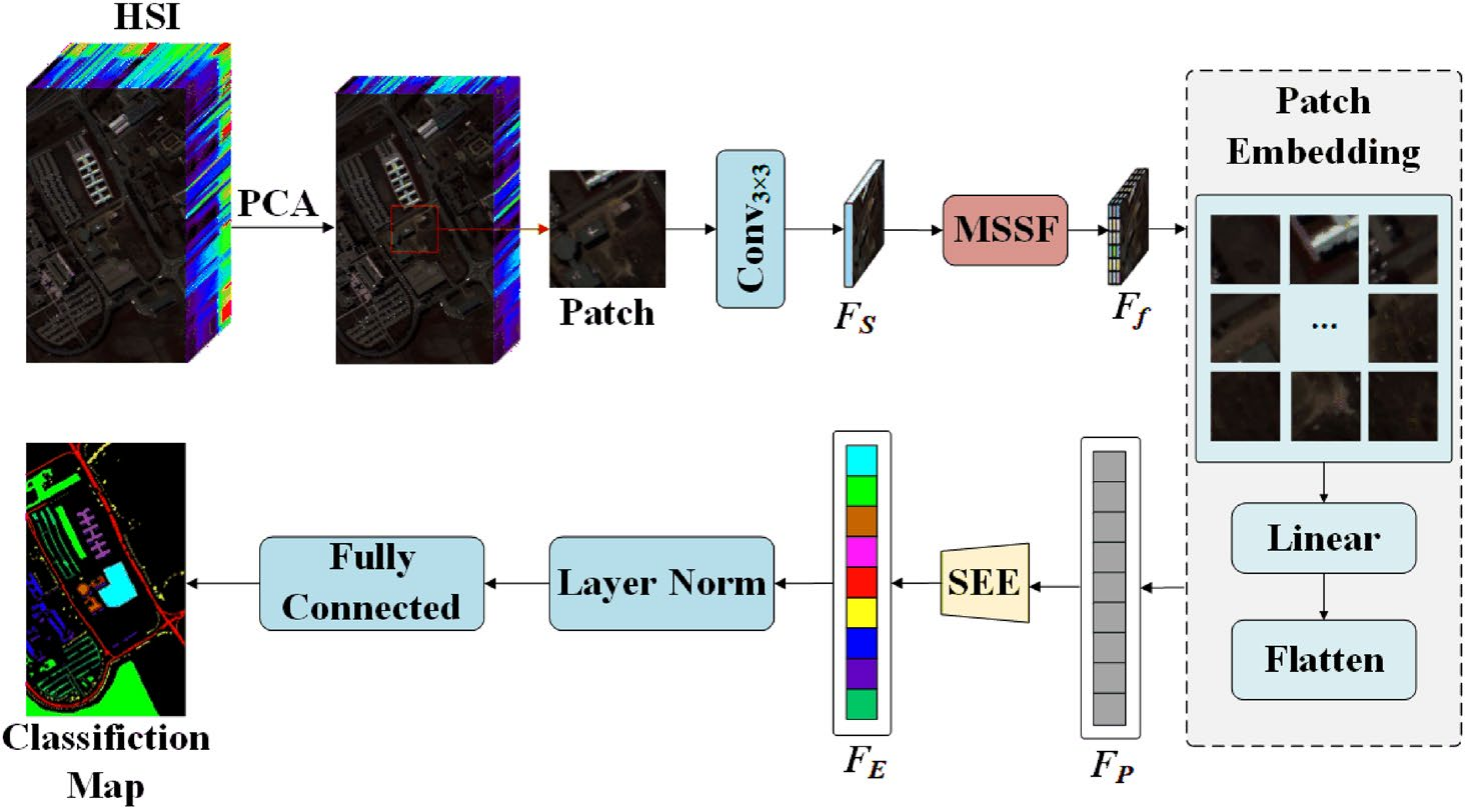
Overall framework of MSNet.

Multiscale spatial-spectral features play a critical role in HSI classification tasks. To fully exploit spatial structural information and spectral variation patterns across different scales, while enhancing the representational capacity and semantic discriminability of features, a novel network architecture based on MSSF and SEE is proposed. The overall framework of the proposed MSNet is illustrated in Fig. 1.

Principal Component Analysis (PCA)^40^ is first applied to the HSI for dimensionality reduction, aiming to retain import spectral information and suppress noise. Based on the reduced-dimensional feature data, a series of spatial patches are generated to serve as input to the subsequent network. A 3$\times$3 convolutional operation is subsequently employed for feature extraction, yielding shallow representations denoted as $F_S$. $F_S$ are then fed into MSSF to perform extraction and fusion of multiscale spatial and spectral features, thereby obtaining the fused feature $F_f$. This process enhances the representational capacity of joint spatial-spectral features, thus improving the classification performance of the model. After being processed by the patch embedding, the features are subsequently fed into the SEE to enhance their semantic representation, thereby facilitating more discriminative feature learning. Finally, the enhanced features are mapped to the target categories through a layer normalization followed by a fully connected layer, thereby achieving precise HSI classification.

### Multiscale spatial-spectral fusion

To address the representational challenges posed by the scale diversity of ground objects in HSI and leverage their multiscale characteristics, the MSSF module is designed. The MSSF module explicitly models the interactions and complementary relationships between features at different scales, thereby effectively mitigating the limitations of fixed-receptive-field methods, which often cause a loss of fine-grained details or contextual information. This significantly enhances the model’s capability to represent complex land-cover structures.

**Fig. 2:**
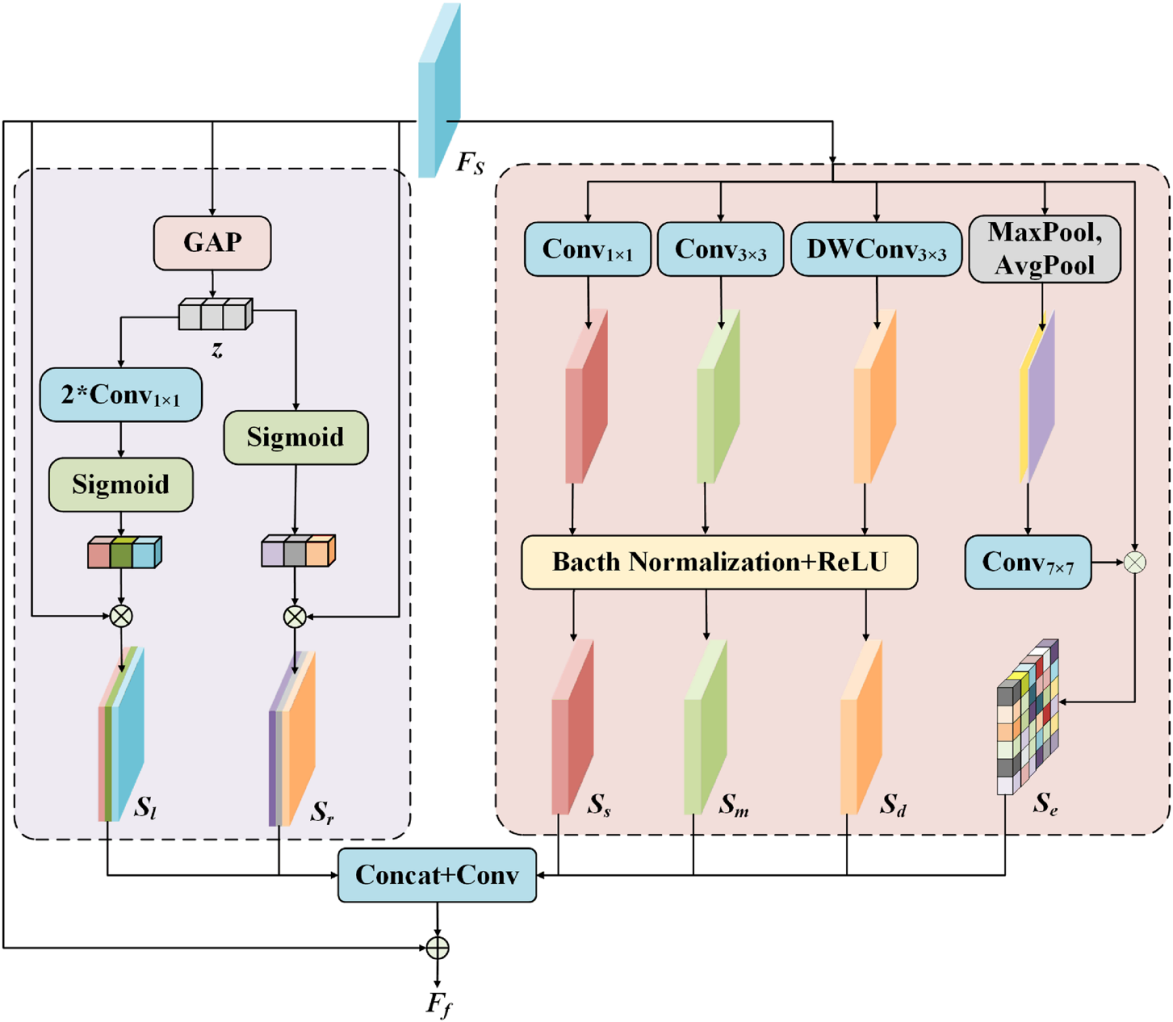
The architecture of MSSF.

The architecture of MSSF is shown in Fig. 2. MSSF takes shallow features $F_S$ $\in$ $R^{C \times H \times W}$ as input, extracts features through the spectral and spatial branches respectively, and outputs the enhanced features $F_f$ after final fusion. The spectral branch first employs Global Average Pooling (GAP)^41^ to aggregate global spectral information. This operation compresses the input feature map $F_S$ into a channel-wise descriptor vector *z*, where the *c*-th element of *z* is computed as:

1 $$\begin{aligned} z_c = \frac{1}{H \times W} \sum _{i=1}^H \sum _{j=1}^W F_S(c, i, j) \end{aligned}$$

where *H* and *W* denote the width and height of $F_S$. The vector *z* is then processed through two consecutive 1$\times$1 convolutional layers and a sigmoid activation function in the left branch to generate the spectral attention weight $Att_l$. Simultaneously, the right branch directly produces the spectral attention weight $Att_r$ via a sigmoid function. The shapes of both $Att_l$ and $Att_r$ are (64, 1, 1). The compuational process can be expressed as follows:

2 $$\begin{aligned} Att_{l}= & \sigma (Conv_{1 \times 1}(Conv_{1 \times 1}(z))) \end{aligned}$$

3 $$\begin{aligned} Att_r= & \sigma (z) \end{aligned}$$

4 $$\begin{aligned} \sigma (x)= & \frac{1}{1 + e^{-x}} \end{aligned}$$

where $\sigma$ denotes the sigmoid function and *Conv* represents the convolutional operation. The enhanced spectral features $S_l$ and $S_r$ are obtrained by performing matrix multiplication between $Att_l$, $Att_r$ and $F_S$, respectively.

**Fig. 3:**
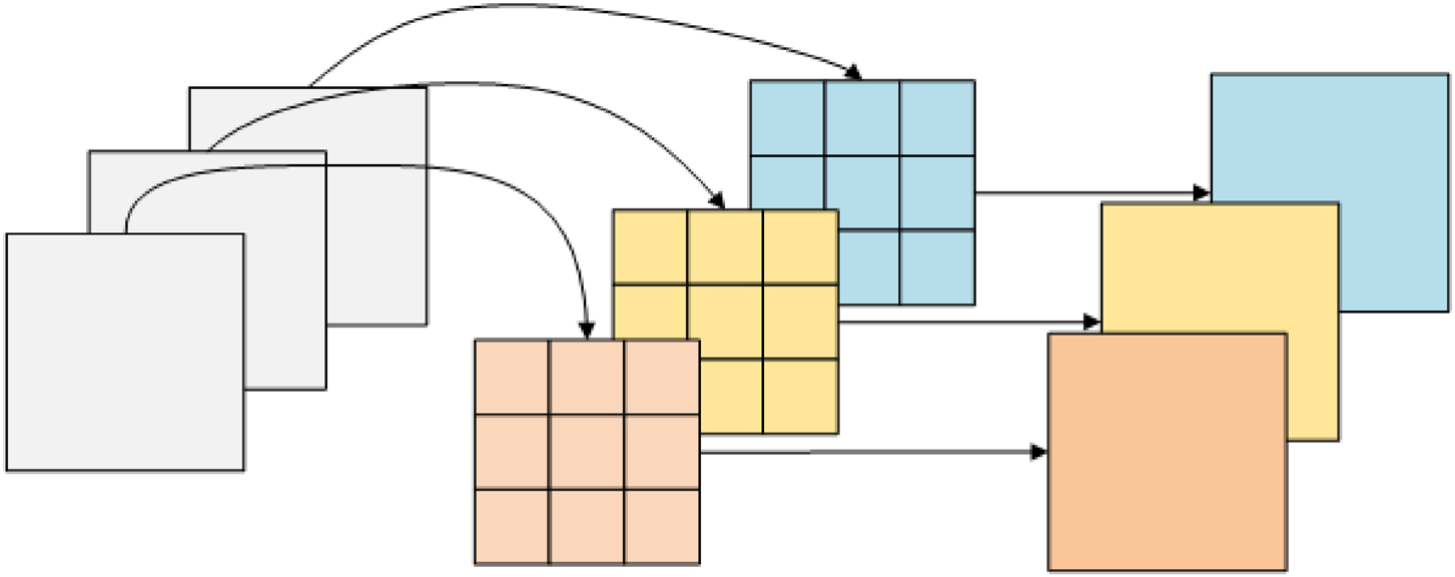
Convolutional process of DwConv.

The spatial branch employs 1$\times$1 and 3$\times$3 convolutional kernels to extract multi-scale spatial features. After processing with batch normalization^42^ and Rectified Linear Unit (ReLU)^43^ activation function, the resulting feature representations are denoted as $S_s$ and $S_m$, respectively. Depthwise Convolution (DwConv)^44^ achieves decoupled spatial feature extraction across spectral channels by maintaining weight independence along the spectral dimension while performing filtering operations in the spatial domain. This operation enables the model to independently capture spatial information within individual spectral channels, thereby significantly enhancing the representational capacity for spatial structures while preserving the completeness of the spectral dimension. The features obtained from applying DwConv to $F_S$ are subsequently processed through batch normalization and ReLU activation function, resulting in the feature representation $S_d$. The convolutional process of DwConv is illustrated in Fig. 3.

The discriminative representation of spatial features remains a core challenge in HSI classification. To enhance the capacity of model for perceiving and representing discriminative spatial structures, an attention mechanism is introduced to achieve adaptive focus on critical regions. We first employ max pooling and average pooling to aggregate spatial information. The pooled features are then concatenated and processed through a 7$\times$7 convolutional layer to generate the spatial attention weight $Att_s$of shape (1, 15, 15). The spatially enhanced feature $S_e$ is obtained by matrix multiplication between $Att_s$ and $F_S$. The computational procedure can be expressed by Equations (5) and (6).

5 $$\begin{aligned} Att_s= & Conv_{7 \times 7}(Concat(MaxPool(F_S), AvgPool(F_S))) \end{aligned}$$

6 $$\begin{aligned} S_{e}= & F_{S} \otimes Att_{s} \end{aligned}$$

where *MaxPool* denotes max pooling, and *AvgPool* represents average pooling. $\otimes$ indicates element-wise multiplication, and *Concat* refers to channel-wise concatenation. Finally, all feature maps are concatenated along the channel dimension, and a convolutional layer is applied to adjust the channel number to *C*, resulting in the fused feature representation $F_f$.

### Semantic enhancement encoder

**Fig. 4:**
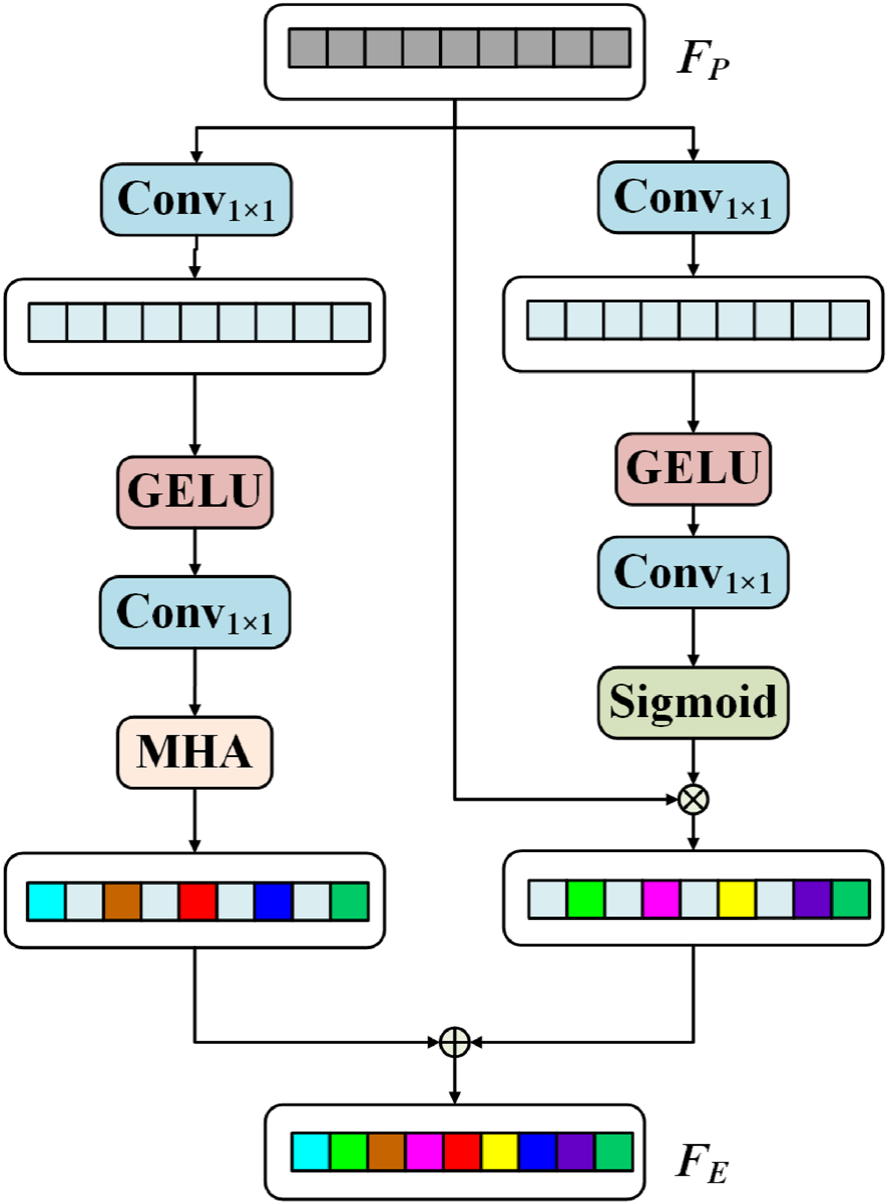
Schematic diagram of the proposed SEE.

Due to the high intra-class variability and low inter-class discriminability inherent in hyperspectral data, the extracted features may contain substantial spectral-spatial uncertainties. To address this issue, SEE incorporates a multi-head attention (MHA)^45^ mechanism to model contextual relationships, effectively capturing long-range dependencies across both spatial and spectral dimensions. The schematic diagram of the proposed SEE is illustrated in Fig. 4.

The proposed SEE employs a dual-branch parallel architecture designed to achieve synergistic enhancement of local details and global contexts through heterogeneous feature extraction pathways. The left branch of SEE is dedicated to global contextual semantic modeling. This pathway utilizes Gaussian Error Linear Units (GELU)^46^ for nonlinear feature transformation and incorporates MHA to capture long-range contextual dependencies. GELU can be mathematically represented by Equation (7).

7 $$\begin{aligned} \text {GELU}(x) = x \Phi (x) \end{aligned}$$

where $\Phi (x)$ is the cumulative distribution function of the standard normal distribution.

**Fig. 5:**
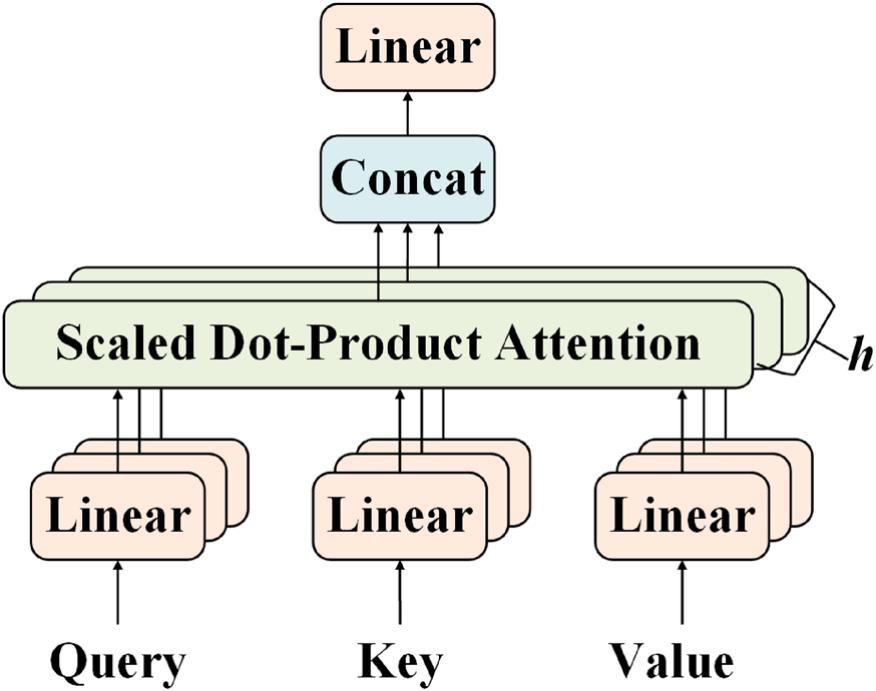
The structure of MHA.

The structure of the MHA is illustrated in Fig. 5. The MHA employs 8 heads with a per-head dimension of 8, and neither positional encodings nor temperature scaling is applied. It projects input features into multiple distinct subspaces through parallel attention heads, thereby capturing diverse dependency patterns among elements. Each head independently computes its query, key, and value representations, focusing on contextual relationships at different semantic levels. The outputs of all heads are concatenated and linearly projected into a unified feature representation. The computational procedure of MHA is defined by Equations (8) and (9).

8 $$\begin{aligned} \text {MHA}(Q, K, V)= & \text {Concat}(\text {head}_1, \ldots , \text {head}_h) W^O \end{aligned}$$

9 $$\begin{aligned} \text {head}_i= & \text {Attention}(QW_i^Q, KW_i^K, VW_i^V) \end{aligned}$$

where $W^O$ is the weight matrix of the weighted summation, $W_i^Q$, $W_i^K$, and $W_i^V$ denote the weight matrix of the *i*-th attention head, respectively.

The right branch of SEE forms a detail-preserving pathway via two convolutional layers, a GELU activation, and a sigmoid function. This pathway is specifically designed to extract and enhance fine-grained details in HSI features. These enhanced details provide complementary information to the contextual modeling branch. Fusion of these detailed features with the global semantic features from the contextual branch effectively compensates for the loss of local structural information resulting from global context modeling, yielding the feature representation $F_E$.

## Experiment

### Dataset description

**Fig. 6:**
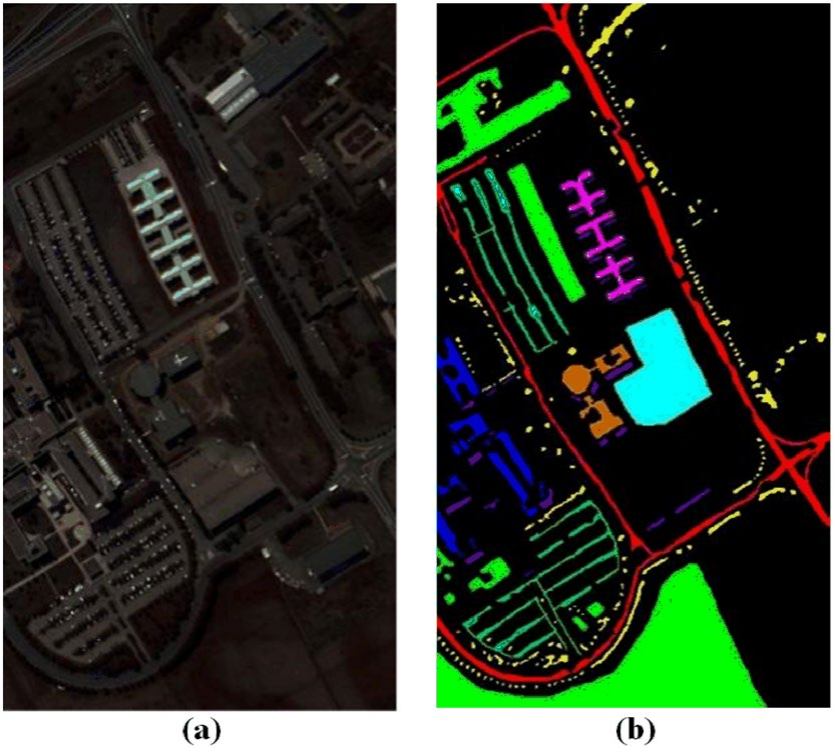
Visualization of PU. (**a**) False color image; (**b**) Ground truth.

Publicly available datasets Pavia University (PU) and Salinas (SA) are adopted for the experimental evaluation. PU dataset was acquired using the reflective optics system imaging spectrometer sensor. It offers a spatial resolution of 1.3 meters per pixel and covers 103 effective spectral bands within the wavelength range of 430–860 nm. The image dimensions are 610$\times$340 pixels, and the dataset includes nine typical land-cover categories such as asphalt, meadows, bare soil, and bricks. Detailed class information is provided in Table 1. Figure 6a depicts the false color image, and Fig. 6b is ground truth.

**Table 1:** Detailed class information of PU.

No	Class	Color	Train	Test	Total
1	Asphalt		20	6611	6631
2	Meadows		20	18,629	18,649
3	Gravel		20	2079	2099
4	Trees		20	3044	3064
5	Metal sheets		20	1325	1345
6	Bare soil		20	5009	5029
7	Bitumen		20	1310	1330
8	Bricks		20	3662	3682
9	Shadows		20	927	947
Total			180	42,596	42,776

**Fig. 7:**
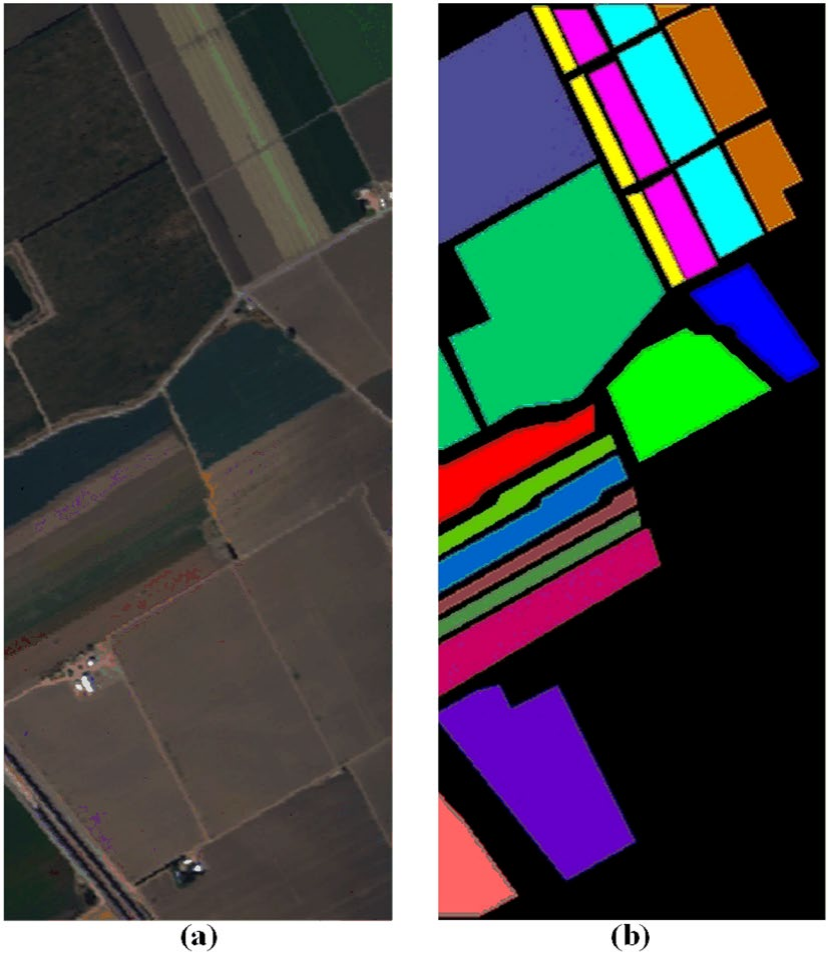
Visualization of SA. (**a**) False color image; (**b**) Ground truth.

SA dataset was collected by the AVIRIS sensor in the Salinas Valley, California. It has a pixel spatial resolution of 3.7 meters per pixel and covers 224 valid spectral bands. The image size is 512$\times$217 pixels, and the dataset contains a total of 16 land-cover categories. Detailed class information and the dataset partition are provided in Table 2. Figure 7a depicts the false color image, and Fig. 7b is ground truth.

**Table 2:** Detailed class information of SA.

No	Color	Land cover type	Train	Test	Total
1		Brocoli_green_weeds_1	20	1989	2009
2		Brocoli_green_weeds_2	20	3706	3726
3		Fallow	20	1956	1976
4		Fallow_rough_plow	20	1374	1394
5		Fallow_smooth	20	2658	2678
6		Stubble	20	3939	3959
7		Celery	20	3559	3579
8		Grapes_untrained	20	11,251	11,271
9		Soil_vinyard_develop	20	6183	6203
10		Corn_senesced_green_weeds	20	3258	3278
11		Lettuce_romaine_4wk	20	1048	1068
12		Lettuce_romaine_5wk	20	1907	1927
13		Lettuce_romaine_6wk	20	896	916
14		Lettuce_romaine_7wk	20	1050	1070
15		Vinyard_untrained	20	7248	7268
16		Vinyard_vertica_trellis	20	1787	1807
Total			320	53,809	54,129

### Experimental setup

The experiment was performed on a computer running the Windows 10 operating system, equipped with the NVIDIA GeForce RTX 3090 GPU. The proposed MSNet was implemented with python 3.8 based on the PyTorch^47^ framework, utilizing key libraries including numpy, scikit-learn, and opencv. The model was optimized using the AdamW optimizer over 200 epochs, with an initial learning rate of 1e-4 and a weight decay coefficient of 1e-5. The hyperparameter settings used in the experiments are provided in Table 3.

**Table 3:** Hyperparameter settings.

Hyperparameter	Value
Initial learning rate	1e−4
Weight decay coefficient	1e−5
Epochs	200
Batch size	128
Optimizer	AdamW

For a comprehensive assessment of the hyperspectral land cover classification model, this study adopts Overall Accuracy (OA), Averagae Accuracy (AA), and the Kappa coefficient (K) as evaluation metrics. These indicators collectively measure classification accuracy and consistency from multiple aspects, rendering them particularly suitable for hyperspectral remote sensing image classification tasks. To ensure statistical reliability, all experiments were conducted with 10 independent runs using random seeds, and the data splits were re-sampled for each run. Only DSFormer and MSNet employ PCA for dimensionality reduction. In our implementation, PCA retains 30 principal components, with input standardization applied beforehand. PCA is fitted exclusively on training pixels during training. The final classification results are reported as the mean accompanied by the standard deviation.

### Effect of patch sizes

To evaluate the effect of input patch size on the performance of MSNet, this study employed five distinct patch sizes measuring 5$\times$5, 10$\times$10, 15$\times$15, 20$\times$20 and 25$\times$25 pixels under identical network architecture, hyperparameter configuration, and training data conditions. A comprehensive evaluation of OA, AA and K was systematically performed to identify the optimal input patch size.

**Table 4:** Classification results under different patch sizes on the PU dataset.

Patch size	OA(%)	AA(%)	K(%)
5$\times$5	91.15 ± 1.58	93.76 ± 0.50	88.50 ± 1.95
10$\times$10	93.91 ± 2.87	96.19 ± 1.25	92.10 ± 3.58
15$\times$15	**95.68 ± 1.78**	**96.58 ± 0.59**	**94.33 ± 2.28**
20$\times$20	94.73 ± 3.51	94.53 ± 2.06	93.12 ± 4.45
25$\times$25	93.26 ± 1.64	91.67 ± 0.85	91.14 ± 2.10

Table 4 presents the performance evaluation results of HSI classification under different patch sizes on the PU dataset, with the best results highlighted in bold. The model achieves optimal performance at a patch size of 15$\times$15, attaining an OA of 95.68%, an AA of 96.58%, and a K of 94.33%. These results demonstrate a significant improvement over those obtained with smaller patch sizes, indicating that a moderate spatial extent can provide more discriminative features for the model. However, classification performance does not increase monotonically with patch size but rather reaches an optimum at a specific scale. When the patch size further increases to 20$\times$20 and 25$\times$25, all performance metrics exhibit a declining trend, with the K dropping to 91.14% at 25$\times$25. This phenomenon indicates that excessively large patches may introduce redundant spatial information or irrelevant background interference, thereby adversely affecting classification performance. In conclusion, the 15$\times$15 patch size is validated as the optimal configuration in this study, achieving the best balance between incorporating sufficient spatial contextual information and mitigating noise introduction.

### Ablation experiment

To evaluate the effectiveness of the SEE and MSSF in HSI classification tasks, this study designed the ablation experiment based on the principle of controlled variables and a progressive module integration strategy. Throughout the experimental process, all models maintained strictly consistent training strategies and hyperparameter configurations to ensure that performance differences were solely attributable to the structural characteristics of the modules themselves, thereby enabling a quantitative and comparable analysis of the functional contributions of each module.

**Table 5:** Ablation experiment of SEE and MSSF on the PU dataset.

Model	SEE	MSSF	OA(%)	AA(%)	K(%)
Ours without SEE and MSSF	$\times$	$\times$	86.94 ± 2.04	89.29 ± 0.66	83.07 ± 2.51
Ours without MSSF	$\checkmark$	$\times$	92.39 ± 2.00	94.45 ± 0.77	90.11 ± 2.52
Ours	$\checkmark$	$\checkmark$	95.68 ± 1.78	96.58 ± 0.59	94.33 ± 2.28

Table 5 presents the ablation experimental results of SEE and MSSF on the PU dataset, where $\checkmark$ indicates the inclusion of the corresponding module and $\times$ indicates its exclusion. The model without both SEE and MSSF achieves an OA of 86.94%, an AA of 89.29% and a K of 83.07%. With the incorporation of the SEE alone, the performance shows significant improvement, reaching 92.39% OA, 94.45% AA, and 90.11% K, demonstrating the effectiveness of the SEE in HSI classification accuracy by enhancing semantic representation. The model with both SEE and MSSF achieves the best performance with 95.68% OA, 96.58% AA, and 94.33% K, indicating that the MSSF further enhances the capability of the model in capturing discriminative features across different scales. The progressive improvement across all three evaluation metrics confirms the individual contributions of both proposed SEE and MSSF to the overall system performance.

**Fig. 8:**
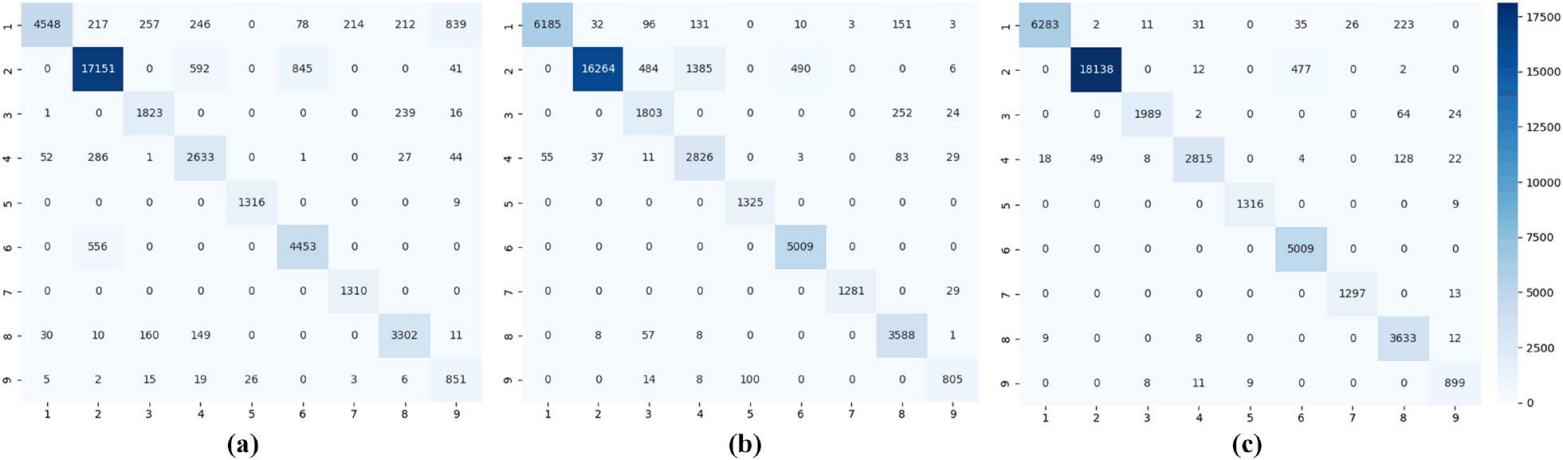
Confusion matrix of ablation experiment. (**a**) Ours without SEE and MSSF; (**b**) Ours without MSSF; (**c**) Ours.

**Fig. 9:**
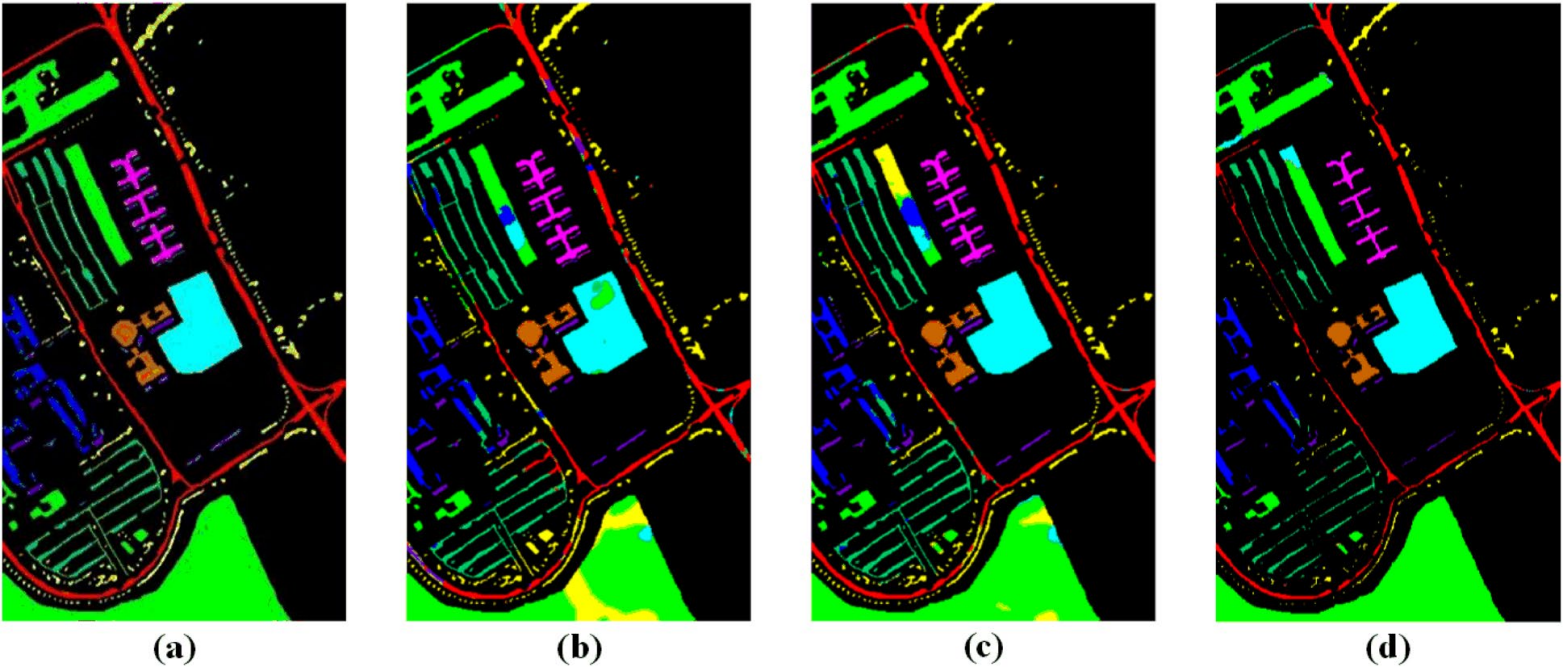
Visualization of ablation experiment. (**a**) Ground truth; (**b**) Ours without SEE and MSSF; (**c**) Ours without MSSF; (**d**) Ours.

Figure 8 presents the confusion matrix corresponding to the ablation experiment. When the model without SEE and MSSF, significant inter-class confusion is observed, particularly among land cover categories with similar characteristics, leading to substantial misclassification. After incorporating the SEE, the diagonal elements of the confusion matrix are notably enhanced, demonstrating that the SEE effectively improves inter-class separability and reduces semantic ambiguity. The model with SEE and MSSF exhibits the clearest diagonal pattern in its confusion matrix, indicating that the MSSF successfully captures spatial information at different scales, thereby further enhancing the classification performance of the model. The visualization results of the ablation experiment, as shown in Fig. 9, provide intuitive validation of the effectiveness of SEE and MSSF in improving HSI classification accuracy.

### Comparative experiment

To validate the performance of the proposed MSNet method, comparative experiments were conducted on the PU and SA datasets with several mainstream models. The selected comparative models include SSFTT, SpectralFormer, GAHT, CAEVT, morphFormer, and DSFormer. The experimental results were evaluated using OA, AA and K metric to compare the classification performance of each model.

**Table 6:** Quantitative result (ACC%±STD%) on the PU dataset.

Class	Methods						
	SpectralFormer	SSFTT	GAHT	CAEVT	morphFormer	DSFormer	MSNet(ours)
1	73.96 ± 7.12	89.21 ± 5.76	89.32 ± 2.39	92.39 ± 4.58	87.13 ± 4.73	**94.92 ± 2.51**	94.49 ± 1.98
2	84.53 ± 6.19	84.42 ± 16.95	90.82 ± 4.83	88.00 ± 6.80	93.80 ± 3.43	90.44 ± 6.33	**94.84 ± 4.39**
3	79.06 ± 8.36	79.72 ± 20.44	96.01 ± 3.86	92.17 ± 4.41	91.15 ± 5.28	94.43 ± 3.91	**96.16 ± 3.19**
4	92.18 ± 1.98	**97.25 ± 1.47**	83.83 ± 3.92	96.13 ± 2.37	85.91 ± 3.08	96.85 ± 1.57	93.71 ± 2.01
5	99.53 ± 0.48	99.73 ± 0.46	95.44 ± 3.71	99.98 ± 0.05	97.64 ± 1.60	**99.99 ± 0.02**	99.71 ± 0.31
6	87.88 ± 4.95	91.30 ± 5.80	97.90 ± 1.07	94.10 ± 4.09	97.95 ± 1.67	98.96 ± 1.13	**99.12 ± 1.12**
7	88.31 ± 7.14	86.44 ± 16.20	95.59 ± 3.46	95.02 ± 1.99	95.85 ± 1.78	99.43 ± 0.49	**99.91 ± 0.14**
8	80.33 ± 7.93	90.50 ± 7.84	95.56 ± 3.04	91.05 ± 3.35	95.47 ± 2.43	93.52 ± 3.81	**95.78 ± 2.17**
9	95.17 ± 2.52	96.86 ± 2.58	85.43 ± 6.17	**99.83 ± 0.20**	86.81 ± 4.69	99.44 ± 0.60	95.49 ± 1.80
**OA(%)**	84.02 ± 2.63	87.99 ± 8.38	91.76 ± 2.01	91.29 ± 2.85	92.73 ± 1.64	93.82 ± 2.64	**95.68 ± 1.78**
**AA(%)**	86.77 ± 1.89	90.60 ± 5.24	92.21 ± 0.90	94.30 ± 0.88	92.41 ± 1.33	96.44 ± 0.63	**96.58 ± 0.59**
**K(%)**	79.39 ± 3.16	84.83 ± 9.94	89.29 ± 2.50	88.72 ± 3.53	90.49 ± 2.10	92.00 ± 3.33	**94.33 ± 2.28**

**Fig. 10:**
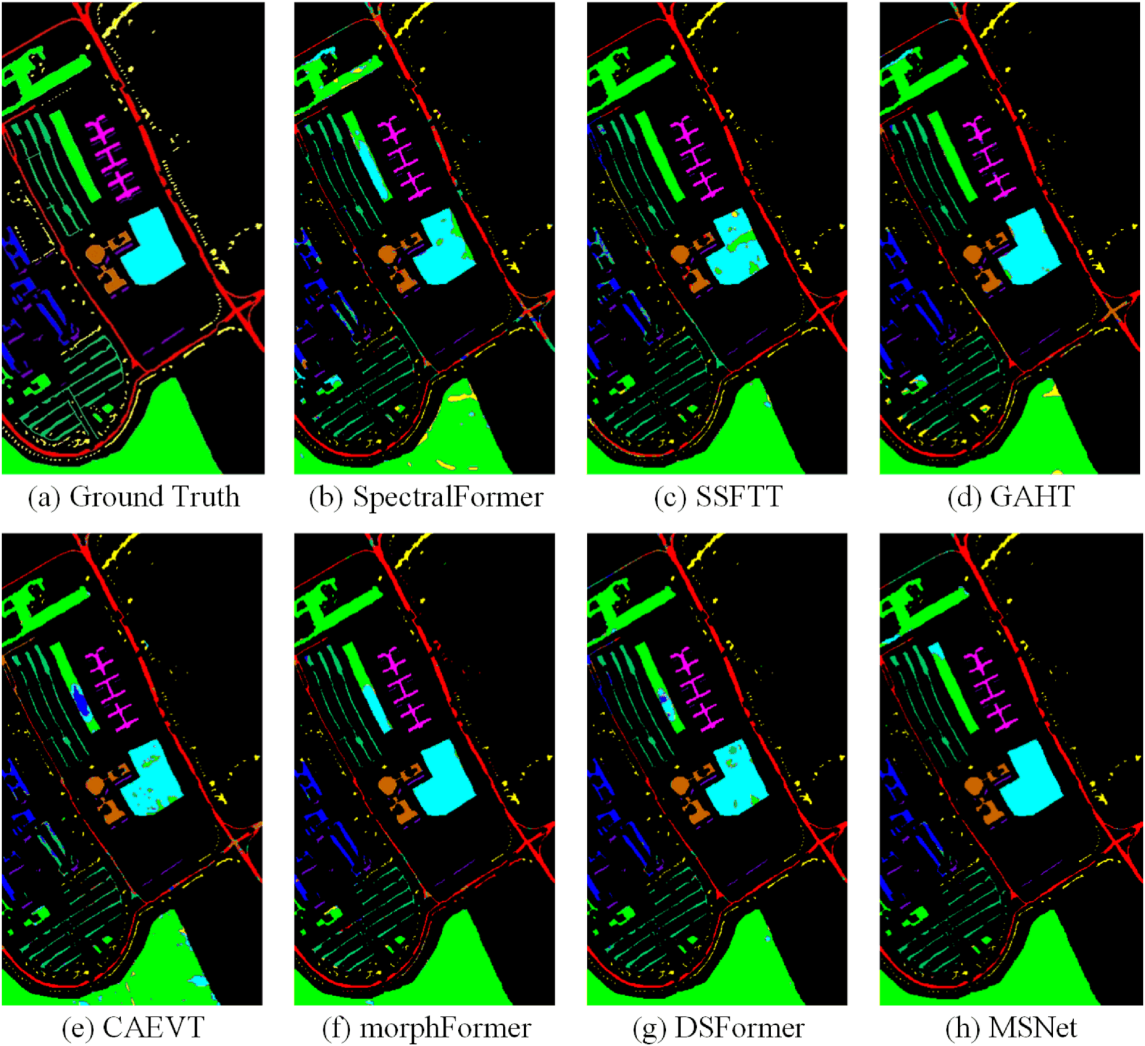
Visualization of comparison experiment on the PU dataset.

The comparative experimental results on the PU dataset are shown in Table 6, with the best results highlighted in bold. Overall, the proposed method achieves the best performance in terms of OA, AA and K, with scores of 95.68%, 96.58% and 94.33%, respectively. These results validate the advancement and effectiveness of our approach. Specifically, our method outperforms all competitors in five out of nine land cover classes. Notably, in challenging categories such as bitumen and bare Soil, MSNet achieved high accuracies of 99.91% and 99.12%, respectively. It delivers dramatic performance improvements in notoriously difficult classes like gravel, where established methods such as SpectralFormer struggle with low scores of 79.06%. This contrast underscores the superior feature representation capability of our method for discriminating complex categories. Although the proposed method does not rank first in a few categories, it still achieves competitive results, with performance gaps within a reasonable margin. Among the comparison methods, DSFormer also demonstrates strong performance, achieving the highest accuracy in two categories and ranking second in OA, AA and K. Some methods such as SSFTT show limited capability in handling complex spatial-spectral relationships, yielding relatively lower OA, AA and K. GAHT, CAEVT and morphFormer exhibit intermediate performance, but our method consistently surpasses them across most categories. In conclusion, the proposed method exhibits superior classification accuracy across most land cover types, validating its effectiveness as a high-performing solution for HSI classification. Furthermore, this superior performance across both common and challenging categories is visually corroborated in Fig. 10, which provides a compelling spatial representation of the classification results.

**Fig. 11:**
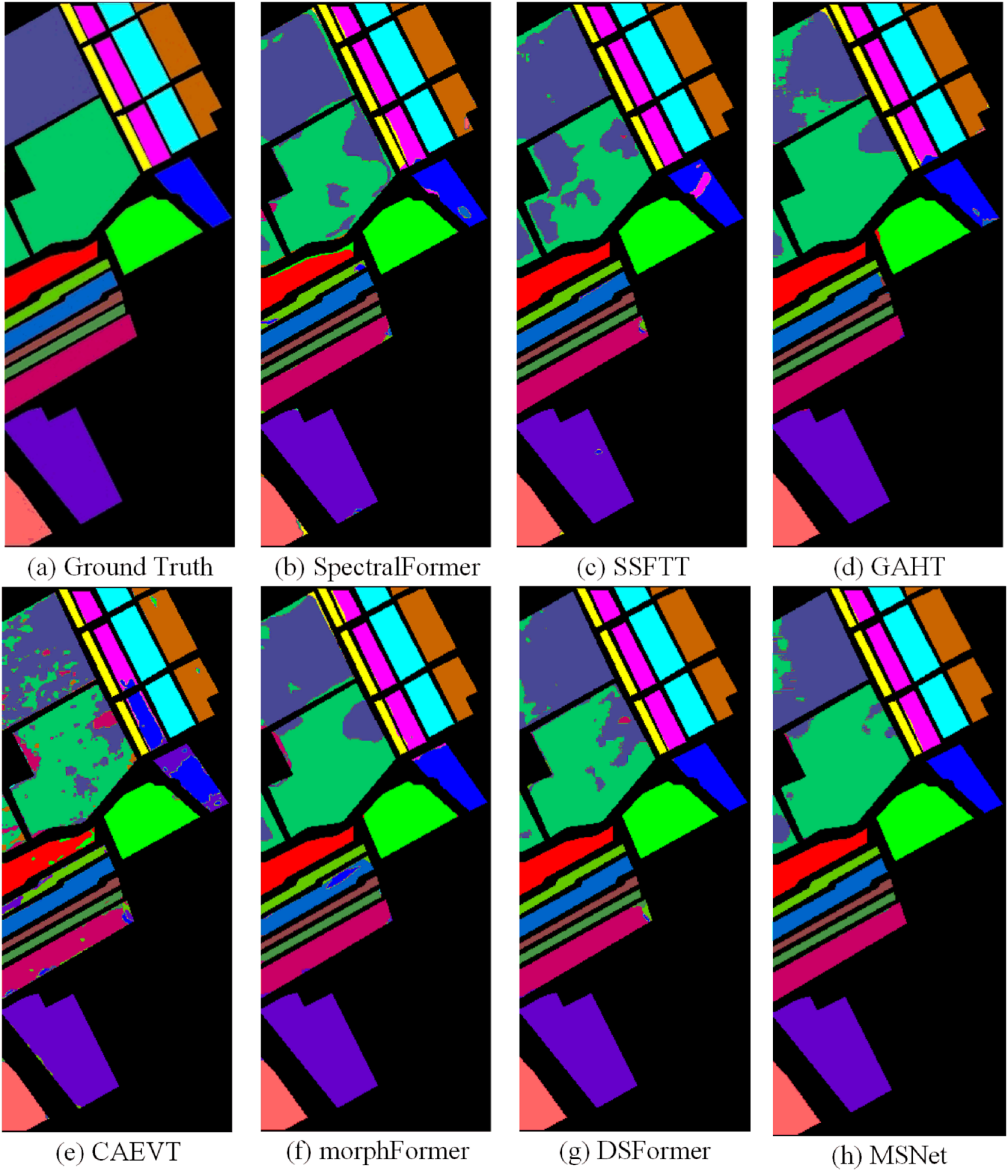
Visualization of comparison experiment on the SA dataset.

The comparative experimental results on the SA dataset are shown in Table 7, with the best results highlighted in bold. As shown, the proposed MSNet achieves outstanding classification performance, attaining the highest OA of 96.84%, AA of 98.65%, and K of 96.48%, outperforming all competing methods. These results firmly establish the superiority and robustness of our approach. Among the comparison methods, DSFormer demonstrates competitive performance, ranking second in OA, AA, and K with scores of 95.77%, 98.24%, and 95.30%, respectively. MorphFormer and GAHT achieve moderate results with OAs of 94.48% and 94.35%, respectively, indicating their reasonable but limited capability in handling the spectral complexity of the Salinas dataset. In contrast, several existing methods exhibit relatively limited performance. SpectralFormer achieves an OA of 91.54%, while SSFTT yields a comparable result of 91.62%. In summary, the experimental results on the SA dataset further validate the superiority of MSNet. Its consistent performance gains across all metrics, confirm its effectiveness as a state-of-the-art solution for hyperspectral image classification. These quantitative findings are further supported by the visual results presented in Fig. 11, which provide additional qualitative evidence of our method’s classification accuracy.

**Table 7:** Quantitative results (ACC% ± STD%) on the SA dataset.

Class	Methods						
	SpectralFormer	SSFTT	GAHT	CAEVT	morphFormer	DSFormer	MSNet(Ours)
1	99.52 ± 0.72	86.46 ± 22.99	97.67 ± 5.51	88.52 ± 21.82	**100**	**100**	**100**
2	95.69 ± 3.44	99.18 ± 1.15	99.78 ± 0.48	98.12 ± 4.70	94.16 ± 12.95	**100**	**100**
3	92.02 ± 4.48	92.62 ± 11.49	91.96 ± 10.75	85.49 ± 12.59	97.86 ± 2.78	99.69 ± 0.52	**99.96 ± 0.06**
4	94.99 ± 3.68	**99.73 ± 0.42**	98.83 ± 2.14	98.76 ± 1.27	98.97 ± 1.32	99.31 ± 0.40	99.73 ± 0.44
5	93.78 ± 2.78	92.41 ± 8.78	97.28 ± 2.68	86.99 ± 14.49	96.02 ± 3.04	**98.14 ± 1.61**	98.03 ± 2.18
6	99.78 ± 0.40	99.56 ± 0.95	99.98 ± 0.03	99.17 ± 1.74	99.00 ± 1.48	**99.99 ± 0.03**	99.79 ± 0.47
7	95.23 ± 3.48	97.92 ± 2.79	99.38 ± 1.33	99.78 ± 0.36	99.26 ± 0.90	**99.95 ± 0.07**	99.91 ± 0.15
8	78.99 ± 9.86	75.38 ± 14.87	81.32 ± 22.58	66.95 ± 14.40	84.31 ± 8.25	86.41 ± 2.57	**90.87 ± 4.98**
9	99.01 ± 0.81	98.58 ± 2.37	99.24 ± 1.35	97.12 ± 2.61	98.39 ± 2.20	99.99 ± 0.01	**100**
10	96.38 ± 1.65	98.03 ± 2.58	97.45 ± 4.27	92.65 ± 3.09	**98.48 ± 1.43**	96.96 ± 1.58	97.98 ± 2.20
11	99.25 ± 0.97	97.00 ± 3.97	98.49 ± 1.95	93.37 ± 12.27	95.66 ± 10.41	**99.99 ± 0.03**	99.98 ± 0.06
12	97.61 ± 1.51	99.29 ± 0.86	98.41 ± 1.57	97.17 ± 2.60	94.31 ± 13.56	99.76 ± 0.33	**99.93 ± 0.03**
13	98.58 ± 1.26	95.28 ± 9.21	99.19 ± 0.63	99.30 ± 0.92	99.41 ± 0.44	99.97 ± 0.10	**100**
14	98.10 ± 2.00	98.95 ± 1.31	98.53 ± 1.69	98.54 ± 1.24	97.01 ± 2.42	99.78 ± 0.29	**99.88 ± 0.19**
15	84.46 ± 15.70	90.02 ± 18.27	94.23 ± 4.96	82.76 ± 8.60	**94.57 ± 5.58**	92.15 ± 2.92	92.67 ± 4.62
16	96.68 ± 5.06	98.73 ± 2.80	**99.92 ± 0.20**	97.71 ± 1.72	98.57 ± 3.71	99.79 ± 0.21	99.75 ± 0.58
**OA(%)**	91.54 ± 1.60	91.62 ± 3.24	94.35 ± 4.47	87.82 ± 2.46	94.48 ± 1.76	95.77 ± 0.47	**96.84 ± 0.91**
**AA(%)**	95.00 ± 0.53	94.95 ± 2.53	96.98 ± 1.55	92.65 ± 1.56	96.62 ± 1.58	98.24 ± 0.23	**98.65 ± 0.38**
**K(%)**	90.60 ± 1.78	90.71 ± 3.58	93.74 ± 4.90	86.50 ± 2.68	93.87 ± 1.95	95.30 ± 0.52	**96.48 ± 1.01**

The computational efficiency of MSNet is evaluated against existing methods in terms of FLOPs and parameter count, as shown in Table 8. MSNet achieves an excellent balance between model complexity and performance, requiring only 46M FLOPs and 168 K parameters. This is significantly more efficient than GAHT which uses 371M FLOPs and 927 K parameters, and SSFTT which uses 123M FLOPs and 484 K parameters, while maintaining superior classification accuracy. Among the compared methods, DSFormer has slightly lower FLOPs of 41M but substantially more parameters of 593 K, over three times that of MSNet. CAEVT achieves comparable FLOPs of 56M but with higher parameters of 206K. In summary, MSNet offers an optimal trade-off between computational efficiency and accuracy, making it well-suited for practical applications with limited resources.

**Table 8:** Computational complexity of various methods.

Method	FLOPs(M)	Param(K)
SpectralFormer	64	232
SSFTT	123	484
GAHT	371	927
CAEVT	56	206
morphFormer	69	149
DSFormer	41	593
MSNet (Ours)	46	168

## Conclusion and future work

This paper proposed a novel HSI classification network based on MSSF and SEE. The experimental results conducted on the Pavia University and Salinas datasets demonstrate that our approach effectively overcomes the limitations of existing methods in extracting discriminative joint spatial-spectral features. The proposed MSSF module successfully captures complementary information across multiple scales through spatial and spectral branches, enabling synergistic feature interaction that significantly enhances the characterization of complex land cover structures with highly variable spectral responses. Furthermore, the incorporation of the multi-head attention mechanism within the SEE module explicitly models global contextual relationships, thereby refining feature representation in semantically critical regions. The outstanding performance of MSNet is quantitatively validated, achieving remarkable OA of 95.68% on the Pavia University dataset and 96.84% on the Salinas dataset, which notably surpasses those obtained by existing mainstream methods. These results conclusively demonstrate the effectiveness and superiority of the proposed architectural design in advancing HSI classification performance. The methodology presented in this work provides a viable and effective solution for remote sensing image analysis tasks, offering significant potential for practical applications in environmental monitoring, agricultural management, and urban planning. Future research will focus on further extending the proposed framework to accommodate more diverse hyperspectral datasets acquired under different conditions, exploring strategies for enhancing computational efficiency to facilitate real-time processing of HSI.

## Supplementary Information

Below is the link to the electronic supplementary material.

Supplementary Information.

## Data Availability

The Pavia University and Salinas datasets used in this study are publicly available in the Hyperspectral Remote Sensing Scenes repository at https://www.ehu.eus/ccwintco/index.php/Hyperspectral_Remote_Sensing_Scenes.

## References

[1] Taghipour, A., Ghassemian, H. Hyperspectral anomaly detection based on frequency analysis of repeated spatial patterns. In *Proceeding of the International Conference on Machine Vision and Image Processing* 1–6 (2020).

[2] Taghipour, A. & Ghassemian, H. Visual attention-driven framework to incorporate spatial-spectral features for hyperspectral anomaly detection. *Int. J. Remote Sens.* **42**(19), 7454–7488 (2021).

[3] Li, S. et al. Deep learning for hyperspectral image classification: An overview. *IEEE Trans. Geosci. Remote Sens.* **57**(9), 6690–6709 (2019).

[4] Lou, C., Al-qaness, M. A., AL-Alimi, D., Dahou, A., Abd Elaziz, M., Abualigah, L., Ewees, A. A. Land use/land cover (LULC) classification using hyperspectral images: A review. *Geo-Spat. inf. Sci.* 28(2), 345-386 (2025).

[5] Farmonov, N. et al. Crop type classification by DESIS hyperspectral imagery and machine learning algorithms. *IEEE J. Sel. Topics Appl. Earth Observ. Remote Sens.* **16**, 1576–1588 (2023).

[6] Ahmad, M. et al. Hyperspectral image classification-Traditional to deep models: A survey for future prospects. *IEEE J. Sel. Topics Appl. Earth Observ. Remote Sens.* **15**, 968–999 (2021).

[7] Camps-Valls, G., Tuia, D., Bruzzone, L. & Benediktsson, J. A. Advances in hyperspectral image classification: Earth monitoring with statistical learning methods. *IEEE Signal Proc. Mag.* **31**(1), 45–54 (2013).

[8] Chang, C. I. Statistical detection theory approach to hyperspectral image classification. *IEEE Trans. Geosci. Remote Sens.* **57**(4), 2057–2074 (2018).

[9] Okwuashi, O. & Ndehedehe, C. E. Deep support vector machine for hyperspectral image classification. *Pattern Recognit.* **103**, 107298 (2020).

[10] Qi, L. et al. Convolutional neural network-based method for agriculture plot segmentation in remote sensing images. *Remote Sens.* **16**(2), 346 (2024).

[11] Liu, D., Zhang, J., Qi, Y., Wu, Y. & Zhang, Y. Tiny object detection in remote sensing images based on object reconstruction and multiple receptive field adaptive feature enhancement. *IEEE Trans. Geosci. Remote Sens.* **62**, 1–13 (2024).

[12] Hamida, A. B., Benoit, A., Lambert, P. & Amar, C. B. 3-D deep learning approach for remote sensing image classification. *IEEE Trans. Geosci. Remote Sens.* **56**(8), 4420–4434 (2018).

[13] Vaswani, A., Shazeer, N., Parmar, N., Uszkoreit, J., Jones, L., Gomez, A. N., Polosukhin, I. Attention is all you need. In *Proceedings of the 31st International Conference on Neural Information Processing Systems.* 6000–6010 (Curran Associates Inc., 2017).

[14] He, X., Chen, Y. & Lin, Z. Spatial-spectral transformer for hyperspectral image classification. *Remote Sens.* **13**(3), 498 (2021).

[15] Liu, Y., Liu, S., Wang, Y., Lombardi, F. & Han, J. A stochastic computational multi-layer perceptron with backward propagation. *IEEE Trans. Comput.* **67**(9), 1273–1286 (2018).

[16] Zhang, X. et al. A lightweight transformer network for hyperspectral image classification. *IEEE Trans. Geosci. Remote Sens.* **61**, 1–17 (2023).

[17] Hong, D., Yokoya, N., Chanussot, J. & Zhu, X. X. An augmented linear mixing model to address spectral variability for hyperspectral unmixing. *IEEE Trans. Image Process.* **28**(4), 1923–1938 (2018). 10.1109/TIP.2018.2878958 30418901

[18] Wang, K. & Yong, B. Application of the frequency spectrum to spectral similarity measures. *Remote Sens.* **8**(4), 344 (2016).

[19] Li, Y., Zhang, H. & Shen, Q. Spectral–spatial classification of hyperspectral imagery with 3D convolutional neural network. *Remote Sens.* **9**(1), 67 (2017).

[20] Yang, X. et al. Hyperspectral image classification with deep learning models. *IEEE Trans. Geosci. Remote Sens.* **56**(9), 5408–5423 (2018).

[21] Roy, S. K., Krishna, G., Dubey, S. R. & Chaudhuri, B. B. HybridSN: Exploring 3-D–2-D CNN feature hierarchy for hyperspectral image classification. *IEEE Geosci. Remote Sens. Lett.* **17**(2), 277–281 (2019).

[22] Zhu, M., Jiao, L., Liu, F., Yang, S. & Wang, J. Residual spectral–spatial attention network for hyperspectral image classification. *IEEE Trans. Geosci. Remote Sens.* **59**(1), 449–462 (2020).

[23] Alkhatib, M. Q. et al. Tri-CNN: A three branch model for hyperspectral image classification. *Remote Sens.* **15**(2), 316 (2023).

[24] Dosovitskiy, A. et al. An image is worth 16x16 words: Transformers for image recognition at scale. In *Proceedings of the International Conference on Learning Representations* (2021).

[25] Yan, H. et al. Hybrid Conv-ViT network for hyperspectral image classification. *IEEE Geosci. Remote Sens. Lett.* **20**, 1–5 (2023).

[26] Hong, D. et al. SpectralFormer: Rethinking hyperspectral image classification with transformers. *IEEE Trans. Geosci. Remote Sens.* **60**, 1–15 (2021).

[27] Mei, S., Song, C., Ma, M. & Xu, F. Hyperspectral image classification using group-aware hierarchical transformer. *IEEE Trans. Geosci. Remote Sens.* **60**, 1–14 (2022).

[28] Yang, X., Cao, W., Lu, Y. & Zhou, Y. Hyperspectral image transformer classification networks. *IEEE Trans. Geosci. Remote Sens.* **60**, 1–15 (2022).

[29] Wang, D., Zhang, J., Du, B., Zhang, L. & Tao, D. DCN-T: Dual context network with transformer for hyperspectral image classification. *IEEE Trans. Image Process.* **32**, 2536–2551 (2023). 37115828 10.1109/TIP.2023.3270104

[30] Huang, Z., Liang, M., Qin, J., Zhong, S., Lin, L. Understanding self-attention mechanism via dynamical system perspective. In *Proceedings of the IEEE/CVF International Conference on Computer Vision* 1412–1422 (2023).

[31] Li, Z. et al. SPFormer: Self-pooling transformer for few-shot hyperspectral image classification. *IEEE Trans. Geosci. Remote Sens.* **62**, 1–19 (2023).

[32] Basha, S. S., Dubey, S. R., Pulabaigari, V. & Mukherjee, S. Impact of fully connected layers on performance of convolutional neural networks for image classification. *Neurocomput.* **378**, 112–119 (2020).

[33] Sun, L., Zhao, G., Zheng, Y. & Wu, Z. Spectral–spatial feature tokenization transformer for hyperspectral image classification. *IEEE Trans. Geosci. Remote Sens.* **60**, 1–14 (2022).

[34] Zhang, Z., Li, T., Tang, X., Hu, X. & Peng, Y. CAEVT: Convolutional autoencoder meets lightweight vision transformer for hyperspectral image classification. *Sensors* **22**(10), 3902 (2022). 35632310 10.3390/s22103902 PMC9146051

[35] Roy, S. K. et al. Spectral–spatial morphological attention transformer for hyperspectral image classification. *IEEE Trans. Geosci. Remote Sens.* **61**, 1–15 (2023).

[36] Zhang, B., Chen, Y., Rong, Y., Xiong, S. & Lu, X. MATNet: A combining multi-attention and transformer network for hyperspectral image classification. *IEEE Trans. Geosci. Remote Sens.* **61**, 1–15 (2023).

[37] Zhao, Z., Xu, X., Li, S. & Plaza, A. Hyperspectral image classification using groupwise separable convolutional vision transformer network. *IEEE Trans. Geosci. Remote Sens.* **62**, 1–17 (2024).

[38] Ahmad, M., Ghous, U., Usama, M. & Mazzara, M. WaveFormer: Spectral-spatial wavelet transformer for hyperspectral image classification. *IEEE Geosci. Remote Sens. Lett.* **21**, 1–5 (2024).

[39] Xu, Y., Wang, D., Zhang, L. & Zhang, L. Dual selective fusion transformer network for hyperspectral image classification. *Neural Netw.* **187**, 107311 (2025). 40048755 10.1016/j.neunet.2025.107311

[40] Greenacre, M. et al. Principal component analysis. *Nat. Rev. Method. Prime.* **2**(1), 100 (2022).

[41] Hsiao, T. Y., Chang, Y. C., Chou, H. H. & Chiu, C. T. Filter-based deep-compression with global average pooling for convolutional networks. *J. Syst. Architect.* **95**, 9–18 (2019).

[42] Ioffe, S., Szegedy, C. Batch normalization: Accelerating deep network training by reducing internal covariate shift. In *Proceedings of the International Conference on Machine Learning* 448–456 (2015).

[43] Ou, S., Guo, Z. & Wang, J. Multistability of fuzzy neural networks with rectified linear units and state-dependent switching rules. *IEEE Trans. Fuzzy Syst.* **31**(5), 1518–1530 (2022).

[44] Guo, Y., Li, Y., Wang, L., Rosing, T. Depthwise convolution is all you need for learning multiple visual domains. In *Proceedings of the AAAI Conference on Artificial Intelligence* 8368–8375 (2019).

[45] Wang, J., Lai, C., Wang, Y. & Zhang, W. EMAT: Efficient feature fusion network for visual tracking via optimized multi-head attention. *Neural Netw.* **172**, 106110 (2024). 38237443 10.1016/j.neunet.2024.106110

[46] Altuntas, B. E., Aksu, O., Celik, M. Y., Durak, M. H. Deep Learning Based Automatic Modulation Recognition Using GELU Activation Function. In *Proceeding of the 4th International Conference on Emerging Smart Technologies and Applications* 1–4 (2014).

[47] Paszke, A. et al. Pytorch: An imperative style, high-performance deep learning library. In *Proceedings of the Advances in Neural Information Processing Systems* 32 (2019).

